# Impact of multifaceted interventions on the knowledge, attitude, and practice of adverse drug reactions reporting among healthcare workers in Vietnam: a comparative intervention study

**DOI:** 10.3389/fphar.2024.1420914

**Published:** 2024-11-08

**Authors:** Hong Tham Pham, Minh-Thy Tran Doan, Thuy Dang Thi, Dung Nguyen Tuan, Minh-Hoang Tran, Thao Ngoc Phuong Nguyen

**Affiliations:** ^1^ Department of Pharmacy, Nguyen Tat Thanh University, Ho Chi Minh City, Vietnam; ^2^ Department of Pharmacy, Nhan Dan Gia Dinh Hospital, Ho Chi Minh City, Vietnam; ^3^ Faculty of Pharmacy, University of Medicine and Pharmacy at Ho Chi Minh City, Ho Chi Minh City, Vietnam; ^4^ NTT Hi-Tech Institute, Nguyen Tat Thanh University, Hồ Chí Minh, Vietnam

**Keywords:** adverse drug reactions, KAP, healthcare workers, multifaceted interventions, Vietnam

## Abstract

**Background:**

Knowledge, attitude, and practice of Adverse Drug Reactions (ADRs) and ADRs reporting among healthcare workers were related to the quality and ADRs reporting rate. The aim of this study was to evaluate the effectiveness of the multifaceted interventions on knowledge, attitude, and practice (KAP) of healthcare workers and to compare the proportion of spontaneous ADRs reports at the study center, before and after instituting multifaceted interventions.

**Methods:**

A comparative intervention study was conducted among healthcare workers at the hospital. The participants were asked to complete a questionnaire before and after instituting the multifaceted interventions to assess the KAP of ADRs and their reporting. The impact of the multifaceted interventions was assessed by comparing their correct responses to the KAP questions and the proportion of spontaneous ADRs reports before and after the interventions. The pre- and post-intervention scores for KAP questions were compared usingMcNemar test by R Language.

**Results:**

388 healthcare workers completed the study. The proportion of participants qualified for ADRs reporting increased significantly, from 73.5% (pre–intervention) to 99.2% (post–intervention) for knowledge scores, from 70.6% to 91.8% for attitude scores, and from 81.4% to 97.2% for practice scores (p < 0.001). Similarly, the number of spontaneous ADRs reports increased by 31% after 3 months of interventions.

**Conclusion:**

Multifaceted interventions instituted at the study center improved the knowledge, attitude, and practice of health workers towards ADRs and spontaneous reporting. It would be beneficial to implement such interventions in other hospitals in Vietnam.

## 1 Introduction

Adverse drug reactions (ADRs) are one of the main causes of morbidity and mortality in developed and developing countries (Bouvy et al., 2015; Angamo et al., 2016). Recently, studies reported that 2%–5% of hospital admissions were caused by ADRs (Brandariz-Núñez et al., 2023; Komagamine, 2024), not a significant decrease compared to the last decade (3.6%–6.3%) (Bénard-Laribière et al., 2015; Angamo et al., 2016). ADRs resulted in 197.000 fatalities each year across the Europe (Bouvy et al., 2015). Furthermore, ADRs significantly increased the economic burden on the society. The healthcare cost due to ADRs ranged from 2,851€ to 9,015€ in the in-patient setting, accompanied by a prolonged length of stay. Similar high costs are associated with ADRs among outpatients with a staggering cost of 174€–8,515€ (Formica et al., 2018). Meanwhile, in France, ADRs have remained an economic burden on the healthcare system since 1998 (Bénard-Laribière et al., 2015).

Pharmacovigilance was defined by the World Health Organization (WHO) as the science and actions connected to the identification, assessment, understanding, and prevention of adverse effects or any other medicine-related problem (WHO, 2012). The pharmacovigilance system collected ADRs reports from national members and submitted them to the WHO database and Vigibase. Several approaches were available to detect ADRs, and spontaneous reporting by healthcare workers is considered easy and cost-effective (WHO, 2014).

Vietnam officially became a member of the WHO program for International Drug Monitoring, which significantly marked the beginning of her pharmacovigilance activities in 1999. Ten years later, the National Drug Information and Adverse Drug Reaction Monitoring Centre was established in Hanoi and same was established in another place, Ho Chi Minh City, in 2011 (Nguyen et al., 2018). Under-reporting that is a major problem of spontaneous ADRs reporting globally (Hanafi et al., 2014; Balan, 2021; El-Dahiyat et al., 2023) is currently undermining pharmacovigilance in Vietnam. The number of reports per million population received annually ranged from 177 to 199 (National Drug Information and Adverse Drug Reaction Monitoring Centre in Vietnam, 2021; 2022), which still unmet the WHO recommended rate of 200 (WHO, 2000). Many studies revealed that the knowledge, attitude, and practice of healthcare workers had impacted the quality and the reporting rate of ADRs (Elkalmi et al., 2014; Balan, 2021; Belhekar et al., 2023). Various strategies such as continuous medical education, financial incentives, improving reporting apps, etc., had been implemented in other countries to promote and improve the knowledge, attitude, and practice (KAP) of healthcare workers towards spontaneous ADRs reporting (Fang et al., 2017; Shchory et al., 2020; Parracha et al., 2023). Given the significant impact of these strategies on healthcare workers, such may be implemented in Vietnam as a means of improving spontaneous ADRs reporting. Nhan Dan Gia Dinh Hospital is one of the first hospitals in Vietnam to apply multifaceted intervention methods to improve KAP for healthcare workers. This study aimed to assess the effectiveness of multifaceted interventions by comparing the KAP of doctors, nurses/midwives, and pharmacists before- and after-intervention. The study also compared the number of spontaneous ADRs reported by healthcare workers in the hospital before and after 3 months of following.

## 2 Methods

### 2.1 Study design

This is a prospective questionnaire-based study involving healthcare workers (doctors, nurses, and pharmacists) working at Nhan Dan Gia Dinh Hospital, Ho Chi Minh City, Vietnam. It is also an interventional study requiring an assessment of the knowledge, attitude, and practice of pharmacovigilance by the participants before- and after- implementation of multifaceted educational interventions. Healthcare workers unwilling to participate or who submitted incompletely filled questionnaires were excluded. The study was conducted in three phases: (i) We assessed the baseline KAP of pharmacovigilance of the participants in December 2022, using a questionnaire adapted from previous studies (Ong The Vu, 2014; Tran Thi Lan Anh, 2017; Le, 2020) (ii) In April 2023, we instituted educational intervention program covering all aspects of pharmacovigilance; (iii) In July 2023, participants were re-assessed using the same questionnaire. Comparisons were made to evaluate the pre- and post-test questionnaire as well as to evaluate the indicators of reporting quantity (total number of spontaneous ADRs reports). Ethical approval of this study was obtained from Nhan Dan Gia Dinh Hospital (Approval number 144 NDGD–HDDD) on 8 November 2022.

### 2.2 Questionnaire

A previously designed questionnaire for the evaluation of the KAP of ADRs and ADRs reporting was adopted and modified (Ong The Vu, 2014; Tran Thi Lan Anh, 2017; Le, 2020). The questionnaire contained four sections. Section 1 collected the demographic characteristics of the participants. Section 2 comprised 14 multi-select multiple-choice questions regarding the knowledge of participants about reporting. Total knowledge scores ranged from 0 to 14, with 1 point for each correct answer and 0 point for the unknown or incorrect answer. Those who scored more than 80% of the total knowledge scores (12 points) were classified as having good knowledge, otherwise having poor knowledge. Section 3 focused on the attitude of reporting using a 3-point Likert scale (2 = “strongly agree,” 1 = “agree,” 0 = “disagree”). This section consisted of 10 items with maximum attitude scores being 20 points. The attitude of the participants was classified as positive and negative based on their total attitude score with the threshold of 16 points (80% of total attitude scores). Section 4 included six multi-select multiple-choice questions regarding the practice of reporting. The correct answer scored 1 point, while the incorrect answer received 0 point. Participants were classified as having good practice if they scored more than 80% of the total practice score (5 points) or poor practice if they scored less than five points. The details of the questionnaire including the correct answer for each question were shown in Supplementary Material 1. Also, we investigated barriers to reporting, factors that discourage participants from reporting, and suggestions to improve reporting.

### 2.3 Interventions

Multifaceted interventions were utilized that included all aspects of management, education, and reporting tools. In terms of management, the promulgation of guidelines on monitoring adverse drug reactions in health establishments made according to Decision No.29/QD–BYT dated 5 January 2022 of the Ministry of Health was delivered to all participants. For the educational interventions, PowerPoint presentations and short lectures were given by researchers on how to recognize ADRs in patients, actions taken and how ADRs were reported. In addition, some activities such as publishing newsletters or documents on drug safety, scientific meetings, and consultations via various forms (e.g., face-to-face interaction, short message services (SMS), phone, and email) were undertaken. Furthermore, an software–based ADR report collection tool was introduced to all participants.

### 2.4 Statistical analysis

Data were entered and analyzed using Microsoft Excel and the R language. The result of the demographic characteristics of the participants were shown as frequency and percentage. The McNemar test was used to compare the effect of the interventions on the KAP of the participants before- and after-implementing the multifaceted interventions. The statistical significance level was considered as 0.05 (p < 0.05). For statistical analysis of the attitude section, responses of “strongly agree” and “agree” were pooled into a single category labeled “Yes,” while “disagree” was labeled as “No.” The indicator of reporting quantity was calculated based on the total number of spontaneous ADRs reported by healthcare workers in the hospital pre- and post-intervention (after 3 months of follow-up).

## 3 Results

### 3.1 Demographics of the participants

Of the 425 healthcare workers interviewed, 388 (91.3%) only completed all the phases of the study. Table 1 showed the demographic characteristics of the participants. The participants were mostly females (73.7%), nurses/midwives (68.3%), and last trained on ADRs reporting a year ago (51.8%).

**TABLE 1:** Demographic characteristics of healthcare workers at Nhan Dan Gia Dinh hospital participated in the study (n = 388).

Characteristics	Frequency (%)
Gender	
Male	102 (26.3)
Female	286 (73.7)
Age groups	
<35 years	196 (50.5)
35–45 years	159 (41.0)
>45 years	33 (8.5)
Professional status	
Doctors	82 (21.1)
Nurses/Midwives	265 (68.3)
Pharmacists	41 (10.6)
Department	
Internal medicine	99 (25.5)
Others	71 (18.3)
Pediatrics	61 (15.7)
Obstetrics and Gynecology	60 (15.5)
Surgery	56 (14.4)
Pharmacy	41 (10.6)
Working experience (years)	
<5 years	79 (20.4)
5–10 years	121 (31.2)
>10 years	188 (48.4)
Period last trained on ADRs reporting	
<1 year	201 (51.8)
1–3 years	124 (32.0)
>3 years	44 (11.3)
None	19 (4.9)

### 3.2 Comparison of participants’ knowledge of ADRs before- and after-interventions

Responses to the knowledge of the health workers about ADRs were compared before- and after-interventions in Table 2. According to question 1, most of the participants were well-educated about the definition of ADRs before the interventions (93.9% of doctors, 86.8% of nurses/midwives, and 95.1% of pharmacists answered correctly). After the interventions, the number of participants gave the correct responses increased to 100%, 95.1%, and 100% for doctors, nurses/midwives, and pharmacists, respectively. Question 2 was about the possible causes of ADRs, the percentage of correct responses pre-test among doctors, nurses/midwives, and pharmacists was quite low (82.9%; 70.2%; 85.4%, respectively). However, the percentage post-test increased sharply (98.8%, 98,1%, and 100%, respectively). Almost all participants were aware of their responsibility to report ADRs after the interventions, as illustrated in question 3. Question 4, 5, and six were about the reporting time frames for fatal or life-threatening, serious, and non-serious ADRs. The correct response rates for questions about reporting time frames were statistically significant between pre- and post-test (p < 0.05). Question 7, 8, 9, and 13 were used to examine all aspects of ADRs reporting, namely the method used to submit reports, the purpose and type of ADRs that need to be reported, as well as the required information that should be included in the report. As shown in Table 2, there was a positive trend in correct response rates after interventions among three groups of participants. As illustrated in question 10, knowledge of where to keep the ADRs report was 100% in both the pre- and post-test. In terms of the management process in question 11, 12, and 14, the percentage of participants informed of this information increased obviously after the interventions, especially for doctors and nurses/midwives. In general, there was a statistically significant improvement in the overall level of good knowledge from 73.5% to 99.2% after the interventions (p < 0.001) (Table 5).

**TABLE 2:** Comparison of knowledge about ADRs and ADRs reporting pre- and post-test among participants.

	Knowledge items	Doctors (n = 82)		p-value*	Nurses/Midwives (n = 265)		p-value*	Pharmacists (n = 41)		p-value*
		Pre-test correct response (n, %^a^)	Post-test correct response (n, %^a^)		Pre-test correct response (n, %^a^)	Post-test correct response (n, %^a^)		Pre-test correct response (n, %^a^)	Post-test correct response (n, %^a^)	
1	Definition of ADRs	77 (93.9)	82 (100.0)	—	230 (86.8)	252 (95.1)	**< 0.001**	39 (95.1)	41 (100.0)	—
2	Possible causes of ADRs	68 (82.9)	81 (98.8)	**< 0.001**	186 (70.2)	260 (98.1)	**< 0.001**	35 (85.4)	41 (100.0)	—
3	ADRs reporting responsibility	80 (97.6)	82 (100.0)	—	235 (88.7)	265 (100.0)	—	39 (95.1)	41 (100.0)	—
4	Reporting time frames for fatal or life-threatening unexpected ADRs	63 (76.8)	76 (92.7)	**< 0.001**	164 (61.9)	243 (91.7)	**< 0.001**	32 (78.0)	39 (95.1)	**0.046**
5	Reporting time frames for all other serious, unexpected ADRs	64 (78.0)	75 (91.5)	**0.015**	161 (60.8)	240 (90.6)	**< 0.001**	33 (80.5)	40 (97.6)	**0.023**
6	Reporting time frames for non-serious ADRs	63 (76.8)	78 (95.1)	**< 0.001**	185 (69.8)	255 (96.2)	**< 0.001**	34 (82.9)	40 (97.6)	**0.041**
7	Method used to submit ADRs reports	65 (79.3)	81 (98.8)	**< 0.001**	200 (75.5)	265 (100.0)	—	36 (87.8)	41 (100.0)	—
8	Purpose of ADRs reporting	71 (86.6)	81 (98.8)	**0.004**	227 (85.7)	264 (99.6)	**< 0.001**	40 (97.6)	41 (100.0)	—
9	Types of ADRs need to be reported	75 (91.5)	82 (100.0)	—	235 (88.7)	265 (100.0)	—	37 (90.2)	41 (100.0)	—
10	Where to keep the ADRs reporting forms	82 (100.0)	82 (100.0)	—	265 (100.0)	265 (100.0)	—	41 (100.0)	41 (100.0)	—
11	Organizations developed the current ADRs forms	56 (68.3)	76 (92.7)	**< 0.001**	132 (49.8)	257 (97.0)	**< 0.001**	28 (68.3)	40 (97.6)	**0.001**
12	Aware of “National guideline of pharmacovigilance” issued by Ministry of Health	72 (87.8)	81 (98.8)	**0.016**	224 (84.5)	265 (100.0)	—	38 (92.7)	41 (100.0)	—
13	The minimum information required for an ADRs report	80 (97.6)	82 (100.0)	—	244 (92.1)	265 (100.0)	—	41 (100.0)	41 (100.0)	—
14	Organization responsible for receiving ADRs reports	68 (82.9)	79 (96.3)	**0.010**	207 (78.1)	261 (98.5)	**< 0.001**	36 (87.8)	41 (100.0)	—

*p < 0.05 by using McNemar test. Bold value indicate statistically significant differences.

^a^ Percentages calculated out of total number of each participant group to each question.

### 3.3 Comparison of participants’ attitude of ADRs reporting before- and after-interventions

To assess the attitude of participants toward ADRs reporting, 10 questions were designed using a 3-point Likert scale (Table 3). Almost all responders showed a positive attitude when considering the risk of ADRs during treatment (100% agreed and totally agreed before- and after-interventions). Only a small proportion of doctors (1.2%) and nurses/midwives (0.8%) disagreed to determine the seriousness of ADRs. Determining the seriousness of ADRs was important in order to suggest appropriate treatment. Also, 2.3% of nurses/midwives did not update their knowledge and expertise about ADRs reporting. Fortunately, all understood the importance of categorizing ADRs and acquired knowledge and expertise about ADRs reporting after the intervention (100% agreed and totally agreed). Nearly 10% of nurses/midwives disagreed that information on ADRs has an impact on the regiments during the treatment of patients before the interventions. After the interventions, all of the responders believed in that. Additionally, the percentage of responders disagreed that healthcare workers should consult with colleagues about the causal relationship between a drug and a drug interaction as well as share experiences about ADR reporting decreased to zero after the intervention. Similarly, the number of participants agreed that reporting ADRs is a professional obligation increased to 100% after the interventions (from 100%, 97.4%, 100% to 100%, 100%, 100% for doctors, nurses/midwives, and pharmacists, respectively). Overall, the rate of participants who had a positive attitude changed significantly before and after exposure to the interventions (p < 0.001) (Table 5).

**TABLE 3:** Comparison of attitude about ADRs and ADRs reporting pre- and post-test among participants.

	Attitude items	Doctors (n = 82)		p-value*	Nurses/Midwives (n = 265)		p-value*	Pharmacists (n = 41)		p-value*
		Pre-test “Yes” ^b^ response (n, %^a^)	Post-test “Yes” ^b^ response (n, %^a^)		Pre-test “Yes” ^b^ response (n, %^a^)	Post-test “Yes” ^b^ response (n, %^a^)		Pre-test “Yes” ^b^ response (n, %^a^)	Post-test “Yes” ^b^ response (n, %^a^)	
1	The risk of ADRs during treatment should be considered	82 (100.0)	82 (100.0)	—	258 (97.4)	265 (100.0)	**0.023**	41 (100.0)	41 (100.0)	—
2	The ADRs reporting forms should be pursuant to the “National guideline of pharmacovigilance”	82 (100.0)	82 (100.0)	—	262 (98.9)	265 (100.0)	0.248	41 (100.0)	41 (100.0)	—
3	Healthcare workers should follow the ADRs reporting time frames	82 (100.0)	82 (100.0)	—	262 (98.9)	265 (100.0)	0.248	41 (100.0)	41 (100.0)	—
4	Healthcare workers should have knowledge and expertise to report ADRs	82 (100.0)	82 (100.0)	—	259 (97.7)	265 (100.0)	**0.041**	41 (100.0)	41 (100.0)	—
5	Healthcare workers should determine the seriousness of ADRs to decide further action taken	81 (98.8)	82 (100.0)	1	263 (99.2)	265 (100.0)	0.480	41 (100.0)	41 (100.0)	—
6	Providing information on ADRs has an impact on the treatment regiments	82 (100.0)	82 (100.0)	—	239 (90.2)	265 (100.0)	**< 0.001**	41 (100.0)	41 (100.0)	—
7	Healthcare workers should consult with colleagues about assessing the causal relationship between an ADRs and medicine before reporting	82 (100.0)	82 (100.0)	—	251 (94.7)	265 (100.0)	**0.001**	41 (100.0)	41 (100.0)	—
8	Healthcare workers should share experiences about ADRs reporting with colleagues	82 (100.0)	82 (100.0)	—	257 (97.0)	265 (100.0)	**0.013**	41 (100.0)	41 (100.0)	—
9	Healthcare workers should take note of feedback after submitting the reports	82 (100.0)	82 (100.0)	—	259 (97.7)	265 (100.0)	**0.041**	41 (100.0)	41 (100.0)	—
10	ADRs reporting is a professional obligation	82 (100.0)	82 (100.0)	—	258 (97.4)	265 (100.0)	**0.023**	41 (100.0)	41 (100.0)	—

*p < 0.05 by using McNemar test. Bold value indicate statistically significant differences.

^a^ Percentages calculated out of total number of each participant group to each question.

^b^ Answering “Totally agree” and “Agree” were considered as “Yes” and then analyzed accordingly.

### 3.4 Comparison of participants’ practice of ADRs reporting before- and after-interventions

A total of six questions were used to seek information on the practice of ADRs reporting. Before the interventions, the proportion of the participants who had good practice in managing ADRs ranged from 87.8%–92.7%. This number increased to 97.0%–100% after the interventions. A significant number of participants reported not only serious ADRs but other ADRs as well (85.3%–92.7%). However, after the interventions, the percentage of correct responses was statistically significant only among the doctors and nurses/midwives groups (p = 0.023; p < 0.001, respectively). Interestingly, 100% of pharmacists complied with the ADRs reporting time frames in both pre- and post-test, while there was a significantly difference in the number of doctors and nurses/midwives in following the time frames before- and after-interventions. Regarding the information required for an ADRs report, only a small percentage of doctors and nurses/midwives fully filled necessary information (43.9% and 51.7%, respectively) whereas the rate of pharmacists was higher (90.2%) before the interventions. After the interventions, the rate increased dramatically among doctors and nurses/midwives (87.8%, p < 0.001 and 84.9%, p < 0.001, respectively). Almost 100% of the participants were able to obtain and send the ADRs reports to the responsible units (Table 4). In general, the percentage of participants who had good practice in ADRs reporting before the interventions was found to be 81.4%. The interventions produced a significant increase in practice skills with rise to 97.2% (p < 0.001) (Table 5).

**TABLE 4:** Comparison of practice about ADRs and ADRs reporting pre- and post-test among participants.

	Practice items	Doctors (n = 82)		p-value*	Nurses/midwives (n = 265)		p-value*	Pharmacists (n = 41)		p-value*
		Pre-test correct response (n, %^a^)	Post-test correct response (n, %^a^)		Pre-test correct response (n, %^a^)	Post-test correct response (n, %^a^)		Pre-test correct response (n, %^a^)	Post-test correct response (n, %^a^)	
1	Practice applied with ADRs	72 (87.8)	82 (100.0)	—	245 (92.5)	257 (97.0)	**0.001**	38 (92.7)	40 (97.6)	0.480
2	Types of ADRs were reported	74 (90.2)	81 (98.8)	**0.023**	226 (85.3)	257 (97.0)	**< 0.001**	38 (92.7)	41 (100.0)	—
3	Where ADRs reports were sent to	82 (100.0)	82 (100.0)	—	263 (99.2)	264 (99.6)	1	40 (97.6)	40 (97.6)	—
4	Reporting timing	75 (91.5)	81 (98.8)	**0.077**	214 (80.8)	258 (97.4)	**< 0.001**	41 (100.0)	41 (100.0)	—
5	Where the ADRs reports were obtained	82 (100.0)	82 (100.0)	—	265 (100.0)	265 (100.0)	—	41 (100.0)	41 (100.0)	—
6	Information required for an ADRs report	36 (43.9)	72 (87.8)	**< 0.001**	137 (51.7)	225 (84.9)	**< 0.001**	37 (90.2)	37 (90.2)	1

*p < 0.05 by using McNemar test. Bold value indicate statistically significant differences.

^a^ Percentages calculated out of total number of each participant group to each question.

**TABLE 5:** The percentage of participants qualified for KAP of ADRs and ADRs reporting (n = 388).

Overall level		Pre-test	Post-test	p-value
Knowledge	Good^a^ (n, %)	285 (73.5)	385 (99.2)	**< 0.001**
Attitude	Positive^b^ (n, %)	274 (70.6)	356 (91.8)	**< 0.001**
Practice	Good^c^ (n, %)	316 (81.4)	377 (97.2)	**< 0.001**

Bold value indicate statistically significant differences.

^a^ Good knowledge was defined as the knowledge score no less than 80% of the total knowledge scores (12 points).

^b^ Positive attitude was defined as the attitude score no less than 80% of the total attitude scores (16 points).

^c^ Good practice was defined as the practice score no less than 80% of the total practice scores (5 points).

### 3.5 Barriers to reporting ADRs

**FIGURE 1:**
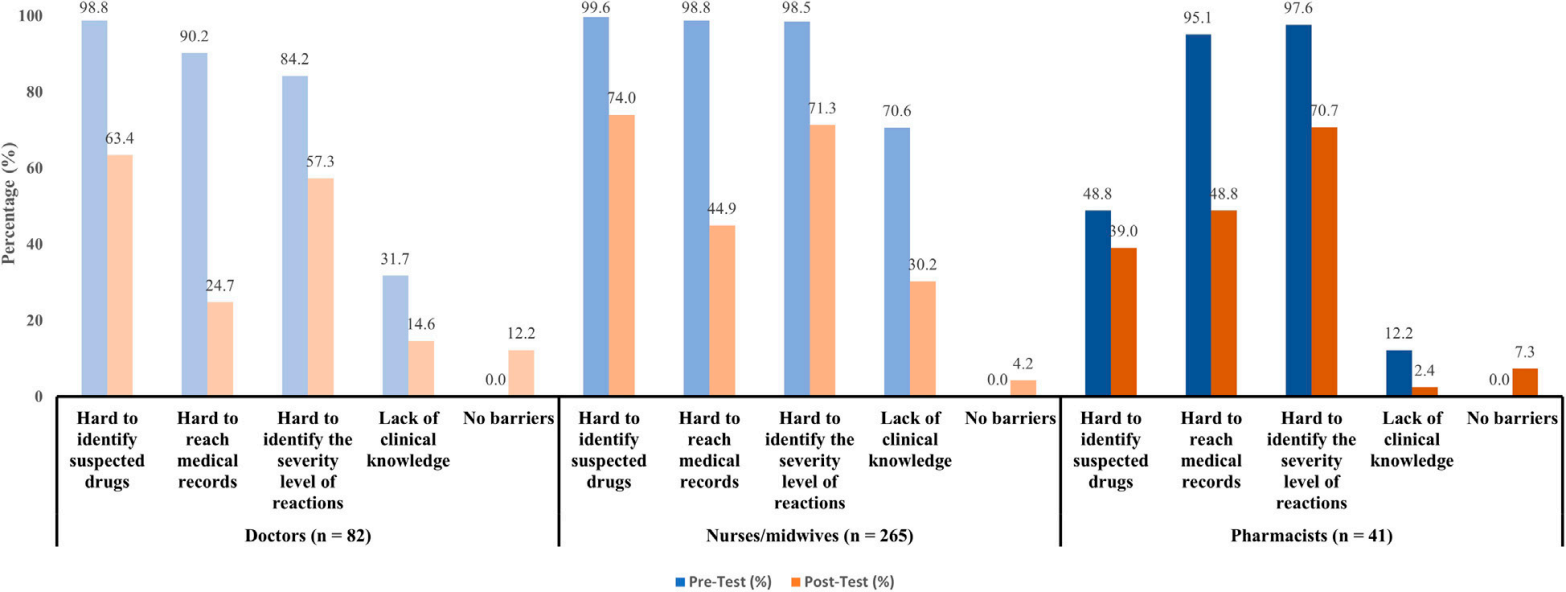
Barriers to ADRs reporting.

Our study also investigated barriers that discouraging participants from reporting ADRs (Figure 1). Prior to the interventions, healthcare workers reported substantial difficulties in reporting ADRs, especially in identifying suspected drugs (94.1%), accessing medical records (96.7%), and determining the severity level of reactions (95.4%). However, the multifaceted interventions led to vastly improvements in reducing these barriers across all professional status (decreased to 68.0%, 41.0%, and 68.3% respectively). The most significant improvements were seen in doctors and nurses/midwives. Pharmacists also showed considerable improvements though they had lower baseline of difficulties. Notably, their clinical knowledge gap narrowed the most after-intervention (only 2.4%). For doctors, 98.8% reported difficulty in identifying suspected drugs, which dropped to 63.4% after-interventions. Barriers in accessing medical records decreased significantly from 90.3% to 24.7%. Before-intervention, 84.2% found assessing the severity level of reactions difficult, and after-intervention, 57.3% still struggled. In terms of clinical knowledge, the data showed a 17.1% improvement. For nurses/midwives, the interventions also benefit these subjects that these barriers improved from 25.6% (difficulty in identifying suspected drugs) to 53.6% (difficulty in identifying the severity level of reactions).

### 3.6 Factors discouraging participants from reporting ADRs

**FIGURE 2:**
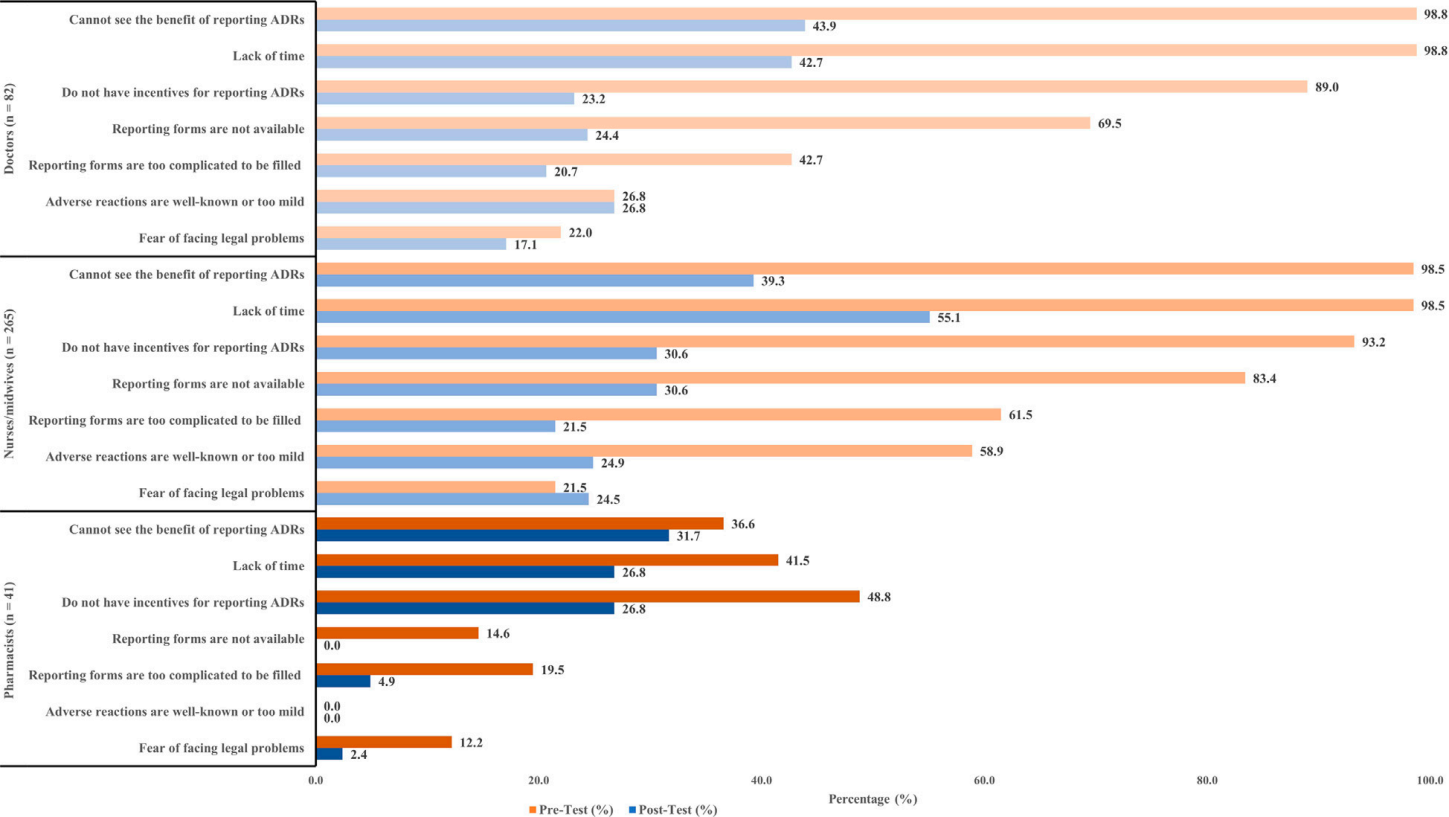
Factors that discourage participants from reporting.

The participants were surveyed about factors that prevented them from reporting an ADRs. Two main factors that discouraged doctors from reporting ADRs include not seeing the benefit of reporting ADRs (98.8%), and lack of time (98.8%). A similar number of nurses/midwives also claimed the same reasons as of doctors (98.5%). However, some pharmacists stated that incentives were a factor that encouraged them to report ADRs (48.8%). The number of participants experiencing discouraging factors tended to decrease significantly after the interventions. Specifically, the issue of timing was notably improved, as the proportion of participants reporting this problem was reduced by half in the post-test compared to the pre-test (Figure 2).

### 3.7 Suggestions on how to improve number and quality of ADRs reports

**FIGURE 3:**
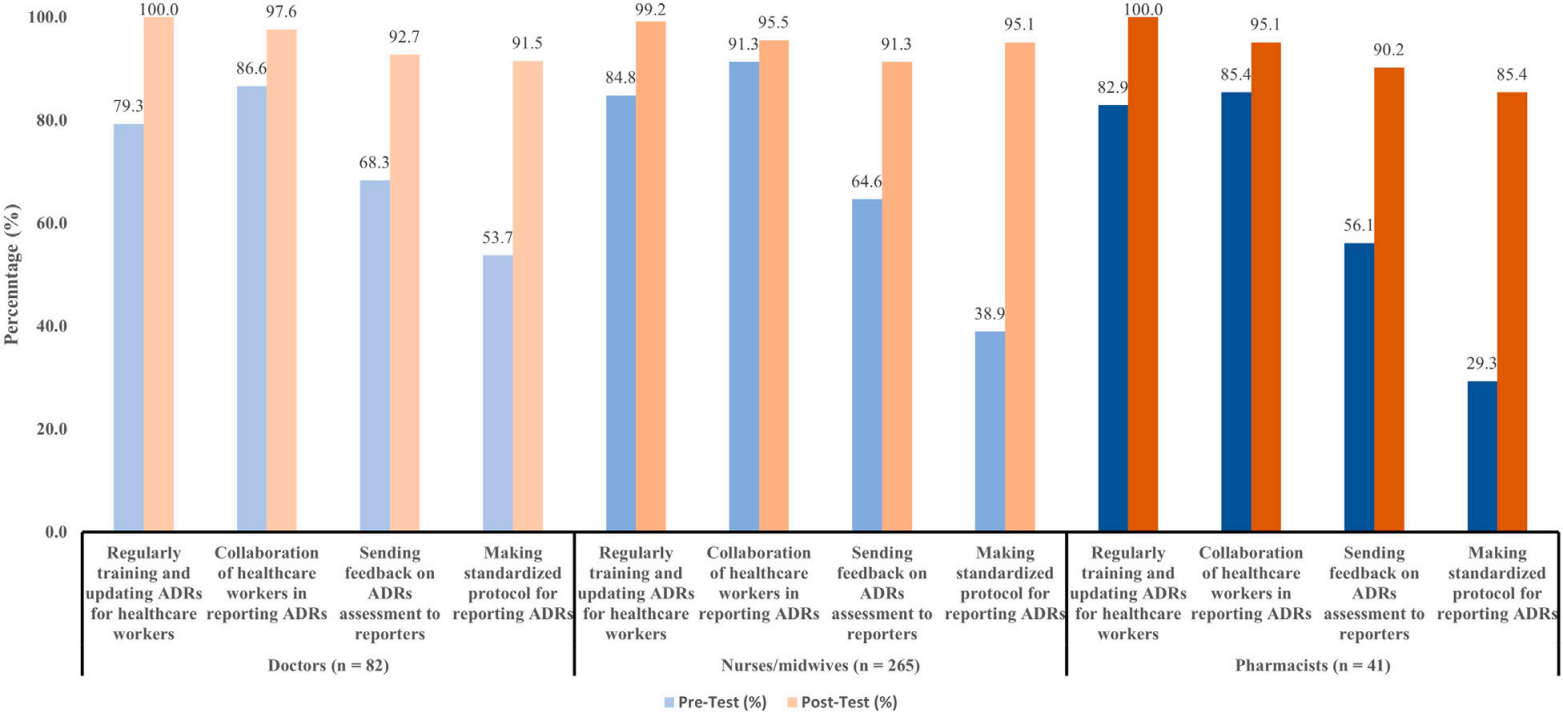
Suggestions to improve numbers and quality of ADRs reports.

Participants were asked to recommend solutions to improve the numbers and quality of ADRs reports. Almost all the doctors and nurses/midwives preferred to be trained and updated on ADRs monitoring and reporting. Additionally, the collaboration of healthcare workers in ADR reporting was also highlighted as an important recommendation. Other recommendations to improve ADRs reports were shown in Figure 3.

### 3.8 Comparison of spontaneous ADRs reports before and after interventions

**FIGURE 4:**
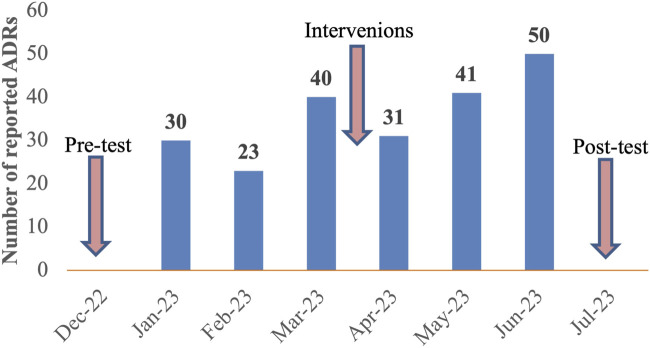
Number of spontaneous ADRs reported before and after 3 months of interventions at Nhan Dan Gia Dinh Hospital from 12th 2022 to 7th 2023.

Three months before the interventions, a total of 93 ADRs were submitted to the ADRs monitoring center with a mean of 31 ADRs per month. After 3 months of interventions, the total number of ADRs increased to 122 with a mean of 41 ADRs per month. Notably, 50 ADRs were reported in the third month following the intervention. Overall, the quantity of ADRs has increased by 31% after the interventions. A detailed overview of the reporting rates before and after interventions was illustrated in Figure 4.

## 4 Discussion

This study evaluated the effect of multifaceted interventions in improving the KAP of doctors, nurses/midwives, and pharmacists, as well as the number of ADRs. Several studies have reported the KAP of healthcare workers towards ADRs and ADRs reporting in Vietnam (Le et al., 2020; Nguyen-Thi et al., 2022; Tran et al., 2023). To the best of our knowledge, this was the first study in Vietnam to implement multifaceted interventions, including delivering guidelines to participants, educational interventions, scientific meetings, consultations, and introducing new online reporting tools. After the interventions, more than 90% of doctors, nurses/midwives, and pharmacists were able to provide the correct responses (Table 2). For the question on types of ADRs that need to be reported during the pre-test, the proportion of participants were of the opinion that all ADRs should be reported was 87.4%. Meanwhile, in Saudi Arabia, the percentage of healthcare professionals reported ADRs to both old and new drugs was the highest (95%) (AlShammari and Almoshen, 2018). The belief that ADRs should be reported for new drugs is a misconception that needs to be clarified in educational materials. The healthcare workers could give the correct to this question increased from approximately 90%–100% after the intervention (Table 2). The study of Genesan also observed the same trend by executing the intervention (Ganesan et al., 2017).

We also observed differences in the rate of knowledge-based correct responses between three groups of participants before the interventions. Among doctors, nurses/midwives and pharmacists, pharmacists had a good level of knowledge about ADRs and ADRs reporting. The results of this study were found to be similar to previous research (Li et al., 2004; Al Rabayah et al., 2019; Shrestha et al., 2020). A previous study showed that a significant factor associated with the knowledge of healthcare workers on ADRs reporting was professional status (Alemu and Biru, 2019). In detail, nurses and physicians were less likely to have adequate knowledge compared to pharmacy professionals (p < 0.05). This could be explained that the curriculums in pharmacovigilance, ADRs and ADRs reporting in universities were inadequately covered in medical training programs in Vietnam (Nguyen-Thi et al., 2022).

Some studies described that good knowledge of ADRs could translate into positive attitude and better rates of reporting (Shchory et al., 2020). After the introduction of the interventions, the attitude of healthcare workers improved. This finding was almost similar to other studies (Ganesan et al., 2017; Shrestha et al., 2020). 100% of doctors, nurses/midwives, and pharmacists accepted that reporting of ADRs was their professional obligation, similar to several studies (Ganesan et al., 2017; AlShammari and Almoshen, 2018; Alshabi et al., 2022) whereas a small proportion of nurses/midwives (2.6%) in pre-test thought that it was the responsibility of doctors and pharmacists, not of them. As obligation was a motive for reporting ADRs (Shchory et al., 2020), the data of our study supported the need to launch a training program to increase the reporting rate.

The interventions also significantly increased the practice of participants, as 98.8% of doctors, 97.4% of nurses/midwives, and 100% of pharmacists reported ADRs immediately when reactions occurred or depending on the severity level of reactions than pre-test (91.5%, 80.8%, and 100% respectively) and very few participants reported at any convenience time. Among all participants surveyed before the interventions, nurses/midwives had the least positive attitude toward ADRs and ADRs reporting, indicating the need for training in this group because nurses provide patient care as well as pay close attention to every detail of patient’s treatment (Tran et al., 2023). The effect of the interventions can be seen in the increase in the number of spontaneous ADRs reports 1 year after the follow-up.

The top barriers to ADRs reporting that doctors, nurses/midwives, and pharmacists pointed out after interventions were the difficulty in identifying suspected drugs and the severity level of reactions. These barriers were also popular reasons that prevented healthcare workers from reporting in previous studies (Hu et al., 2022; Nguyen-Thi et al., 2022). Lacking clinical knowledge could be translated into under-reporting (Kalikar et al., 2020), however these issues were addressed in the interventions, resulting in the number of concerned healthcare workers decreasing sharply in our study as well as few cited no drawbacks in ADRs reporting. These results clearly demonstrated the effort and effect of researchers in training and clarifying the process of reporting.

Under-reporting of ADRs was a major problem in some countries (Ahmad et al., 2013; Agarwal et al., 2013; Hallit et al., 2019). Our study discussed the factors that discourage the reporting of ADRs. “Cannot see the benefit of reporting ADRs,” “Lack of time,” and “Do not have incentives for reporting ADRs” appeared to be the main underlying factors. These factors in our study were also reported in another study in Vietnam (Nguyen-Thi et al., 2022) and other countries (Shrestha et al., 2020; Alshabi et al., 2022). There were several causes of ADRs under-reporting were reported, namely difficulty in deciding whether ADRs have occurred or not (Aziz et al., 2022), lack of instruction for ADRs reporting (Nguyen-Thi et al., 2022), managing patients were more important, patient confidentially issue (Nisa et al., 2018), unavailability of professional environment to discuss ADRs (Alshabi et al., 2022). Regularly training in ADRs and other actions such as collaboration of healthcare workers in reporting, sending feedback on ADRs assessment to reporters, or establishing a standardized protocol for reporting proved to be effective in improving the number and quality of ADRs reports in this study. Our results were in line with other studies in Vietnam (Nguyen-Thi et al., 2022), in Nepal (Shrestha et al., 2020), in India (Ganesan et al., 2017). Furthermore, some recommendations were documented that could be applied in our setting, such as including ADRs forms along with the case sheet, developing mobile apps for reporting, discussing ADRs cases every month during pharmacovigilance meetings, and frequent SMS/email about reporting (Ganesan et al., 2017; Shrestha et al., 2020).

The strengths of our study were that the effectiveness of multifaceted interventions has been demonstrated through the improvement in KAP of ADRs reporting among healthcare workers and the increased number of spontaneous ADRs during the same period following. We also investigated the barriers to ADRs reporting and popular causes of under-reporting among groups of the participants, thereby suggesting the appropriate solutions for each group. However, several limitations could also be addressed. Firstly, we did not recruit all healthcare workers in the hospital. Further research should include variable healthcare workers at different departments; thus, the outcome may be generalizable to other settings. Second, we evaluated the indicator of reporting quantity based on the spontaneous ADRs, which had the disadvantages of incomplete or inaccurate data, and may not detect all types of ADRs (e.g., potential, serious ADRs).

## 5 Conclusion

The results of our study showed that the multifaceted interventions improved the KAP of doctors, nurses/midwives, and pharmacists towards ADRs and ADRs reporting. Therefore, we recommend that similar periodic interventions should be carried out in the study setting, as well as throughout the country.

## Data Availability

The raw data supporting the conclusions of this article will be made available by the authors, without undue reservation.
