# Supplementary material for: Impact of multifaceted interventions on the knowledge, attitude, and practice of adverse drug reactions reporting among healthcare workers in Vietnam: a comparative intervention study
Source: Front Pharmacol. 2024 Nov 8;15:1420914. doi: 10.3389/fphar.2024.1420914 (PMC11581852; doi:10.3389/fphar.2024.1420914)
Supplement: Supplementary file 1 [file DataSheet1.docx]

Supplementary Material

Impact of multifaceted interventions on the knowledge, attitude, and practice of adverse drugs reactions among healthcare workers in Vietnam: a comparative intervention study

Hong Tham Pham, Minh-Thy Tran Doan, Thuy Dang Thi, Dung Nguyen Tuan, Minh-Hoang Tran^*^, Thao Ngoc Phuong Nguyen^*^

*** Correspondence:** Minh-Hoang Tran: [tmhoang@ntt.edu.vn](mailto:tmhoang@ntt.edu.vn); Thao Ngoc Phuong Nguyen: [nnpthao.chdlvdls21@ump.edu.vn](mailto:nnpthao.chdlvdls21@ump.edu.vn)

**The questionnaire**

**Section 1: General information**

**Age**:……………………………………………………………………………………………………...

**Gender**:…………………………………………………………………................................................

**Professional status**:  Doctor  Pharmacist  Nurse / Midwife

**Department**:…………………………………………………………….................................................

**Working experience (years**):…………………………………………………………………………..

**Period last trained on ADRs reporting:**

 Non-participation  < 1 year

 1 - 3 years  > 3 years

**Section 2: ADRs knowledge**

**1. Definition of ADRs:** (Can select multiple answer)

a. A noxious response to drug

b. An unintended response to drug

c. Occurs at doses normally used in man for the prophylaxis, diagnosis, or therapy of disease, or for the modifications of physiological function

d. Unknown

**2. Possible causes of ADRs:** (Can select multiple answer)

a. Drug quality

b. Characteristics of drugs

c. Overdose

d. Drug abuse

e. Using drug without appropriate indication

f. Unknown

**3. ADRs reporting responsibility:** (Can select multiple answer)

a. Doctors

b. Nurses/Midwives

c. Pharmacists

d. Technicians

e. Patients/Consumers

f. Unknown

**4. Reporting time frames for fatal or life-threatening unexpected ADRs:**

a. No later than 7 calendar days

b. No later than 15 calendar days

c. No later than 30 calendar days

**5. Reporting time frames for all other serious, unexpected ADRs:**

a. No later than 7 calendar days

b. No later than 15 calendar days

c. No later than 30 calendar days

**6. Reporting time frames for non-serious ADRs:**

a. No later than 7 calendar days

b. No later than 15 calendar days

c. No later than 30 calendar days

**7. Method used to submit ADRs reports:** (Can select multiple answer)

a. Paper form

b. Website

c. Phone

d. Fax

e. Email

f. Unknown

**8. Purpose of ADRs reporting:** (Can select multiple answer)

a. Detect and identify new ADRs

b. Be part of the work

c. Ensure patient safety

d. Determine frequency of ADRs

e. Share ADRs information with colleagues

f. Unknown

**9. Types of ADRs need to be reported:** (Can select multiple answer)

a. Serious, unexpected ADRs

b. All suspected ADRs

c. All suspected adverse reactions to established drugs

d. All suspected adverse reactions to new drugs

e. Suspected given ADRs

f. Unknown

**10. Where to keep the ADRs reporting forms:** (Can select multiple answer)

a. Department/Faculty of work

b. Planning and Management Department

c. Faculty of Pharmacy

d. National DI & ADR Center

e. Unknown

**11. Organizations developed the current ADRs forms:**

a. Ministry of Health

b. National department of health

c. Hospital

**12. Aware of National guideline on the pharmacovigilance system issued by Ministry of Health:**

a. Yes

b. No

**13. The minimum information required for an ADRs report:** (Can select multiple answer)

a. Patient information

b. Details of suspected ADR(s)

c. Details of suspected medication(s)

d. Reporter and workplace

e. Unknown

**14. Organization responsible for receiving ADRs reports:**

a. Planning and Management Department/ Faculty of Pharmacy

b. Center for Monitoring ADRs in the South (Cho Ray Hospital)

c. Pharmaceutical companies/ Medical representatives

d. Unknown

**Section 3: ADRs atiitude**

**1. The risk of ADRs during treatment should be considered**

 Strongly agree  Agree  Disagree

**2. The ADRs reporting forms should be pursuant to the “National guideline of pharmacovigilance”**

 Strongly agree  Agree  Disagree

**3. Healthcare workers should follow the ADRs reporting time frames**

 Strongly agree  Agree  Disagree

**4. Healthcare workers should have knowledge and expertise to report ADRs**

 Strongly agree  Agree  Disagree

**5. Healthcare workers should determine the seriousness of ADRs to decide further action taken**

 Strongly agree  Agree  Disagree

**6. Providing information on ADRs has an impact on the treatment regiments**

 Strongly agree  Agree  Disagree

**7. Healthcare workers should consult with colleagues about assessing the causal relationship between an ADRs and medicine before reporting**

 Strongly agree  Agree  Disagree

**8. Healthcare workers should share experiences about ADRs reporting with colleagues**

 Strongly agree  Agree  Disagree

**9. Healthcare workers should take note of feedback after submitting the reports**

 Strongly agree  Agree  Disagree

**10. ADRs reporting is a professional obligation**

 Strongly agree  Agree  Disagree

**Section 4: ADRs practice**

**1. Practice applied with ADRs:** (Can select multiple answer)

a. Handle reaction

b. Check all using medications

c. Share information with colleagues

d. Check the appearance of the remaining medication(s)

e. Record clinical and subclinical abnormalities in medical record

**2. Types of ADRs were reported:** (Can select multiple answer)

a. Serious ADRs

b. Suspected ADRs

c. Any ADRs of established drugs

d. Any ADRs occurred

e. ADRs of new drugs

f. Usual ADRs

**3. Where ADRs reports were sent to:** (Can select multiple answer)

a. Faculty of Pharmacy

b. National Drug Information and Adverse Drug Reaction Monitoring Centre/ Center for monitoring ADRs in the South (Cho Ray Hospital)

c. Pharmaceutical companies/ Distribution companies/ Medical representatives

d. National department of health

**4. Reporting timing:** (Can select multiple answer)

a. Immediately when reactions occurred

b. Depends on the severity level of reactions

c. Any convenient time

**5. Where the ADRs reports were obtained:** (Can select multiple answer)

a. Department/Faculty of work

b. Faculty of Pharmacy

c. Planning and Management Department

d. Website of the National Drug Information and Adverse Drug Reaction Monitoring Centre (canhgiacduoc.org.vn)

**6. Information required for an ADRs report:** (Can select multiple answer)

a. Patient information

b. Details of suspected ADR(s)

c. Details of suspected/ concomitant medication(s)

d. Reporter and workplace

e. Prescriber information

**Barriers to ADRs reporting**

 Hard to identify suspected drugs

 Hard to reach medical records

 Hard to identify the severity level of reactions

 Lack of clinical knowledge

 No barriers

**Factors that discourage from reporting**

 Cannot see the benefit of reporting ADRs

 Lack of time

 Do not have incentives for reporting ADRs

 Reporting forms are not available

 Reporting forms are too complicated to be filled

 Adverse reactions are well-known or too mild

 Fear of facing legal problems

**Suggestions to improve numbers and quality of ADRs reports:**

 Regularly training and updating ADRs for healthcare workers

 Collaboration of healthcare workers in reporting ADRs

 Sending feedback on ADRs assessment to reporters

 Making standardized protocol for reporting ADRs

| **No.** | **Correct answers** |
| --- | --- |
| **Section 2: Knowledge** | |
| 1 | a, b, c (1 point for choosing ≥ 2 out of 3 correct answers) |
| 2 | a, b |
| 3 | a, b, c, d, e (1 point for choosing ≥ 3 out of 5 correct answers) |
| 4 | a |
| 5 | b |
| 6 | c |
| 7 | a, b, c, d, e (1 point for choosing ≥ 3 out of 5 correct answers) |
| 8 | a, b, c, d, e (1 point for choosing ≥ 3 out of 5 correct answers) |
| 9 | a, b, c, d, e (1 point for choosing ≥ 3 out of 5 correct answers) |
| 10 | a, b, c, d (1 point for choosing ≥ 2 out of 4 correct answers) |
| 11 | a |
| 12 | a |
| 13 | a, b, c, d (1 point for choosing ≥ 2 out of 4 correct answers) |
| 14 | b |
| **Section 3: Attitude** | |
| 1 | 2 points for choosing “Strongly agree”, 1 point for choosing “Agree”, 0 point for choosing “Disagree” |
| 2 | 2 points for choosing “Strongly agree”, 1 point for choosing “Agree”, 0 point for choosing “Disagree” |
| 3 | 2 points for choosing “Strongly agree”, 1 point for choosing “Agree”, 0 point for choosing “Disagree” |
| 4 | 2 points for choosing “Strongly agree”, 1 point for choosing “Agree”, 0 point for choosing “Disagree” |
| 5 | 2 points for choosing “Strongly agree”, 1 point for choosing “Agree”, 0 point for choosing “Disagree” |
| 6 | 2 points for choosing “Strongly agree”, 1 point for choosing “Agree”, 0 point for choosing “Disagree” |
| 7 | 2 points for choosing “Strongly agree”, 1 point for choosing “Agree”, 0 point for choosing “Disagree” |
| 8 | 2 points for choosing “Strongly agree”, 1 point for choosing “Agree”, 0 point for choosing “Disagree” |
| 9 | 2 points for choosing “Strongly agree”, 1 point for choosing “Agree”, 0 point for choosing “Disagree” |
| 10 | 2 points for choosing “Strongly agree”, 1 point for choosing “Agree”, 0 point for choosing “Disagree” |
| **Section 4: Practice** | |
| 1 | a, b, c, d, e, f (1 point for choosing ≥ 3 out of 6 correct answers) |
| 2 | a, b, c, d, e, f (1 point for choosing ≥ 3 out of 6 correct answers) |
| 3 | a, b, c (1 point for choosing ≥ 1 correct answers) |
| 4 | a, b (1 point for choosing ≥ 1 correct answers) |
| 5 | a, b, c, d (1 point for choosing ≥ 1 out of 4 correct answers) |
| 6 | a, b, c, d |
